# Comparison of the clinical characteristics of SARS-CoV-2 Delta (B.1.617.2) and Omicron (B.1.1.529) infected patients from a single hospitalist service

**DOI:** 10.1186/s12879-023-08714-x

**Published:** 2023-10-31

**Authors:** N. Radhakrishnan, M. Liu, B. Idowu, A. Bansari, K. Rathi, S. Magar, L. Mundhra, J. Sarmiento, U. Ghaffar, J. Kattan, R. Jones, J. George, Y. Yang, F. Southwick

**Affiliations:** 1 Division of Hospital Medicine, Department of Medicine, University of Florida College of Medicine, 6362 NW 41st Ave, Gainesville, FL 32606 USA; 2 Department of Biostatistics, College of Public Health and Health Professions, University of Florida, Gainesville, FL USA; 3 Department of Statistics, Franklin College of Arts and Sciences, University of Georgia, 310 Herty Drive, Athens, GA 30602 Greece

**Keywords:** COVID-19, SARS-CoV-2, Delta variant, Omicron variant, Mortality, Virulence, Clinical manifestations

## Abstract

**Background:**

While existing evidence suggests less severe clinical manifestations and lower mortality are associated with the Omicron variant as compared to the Delta variant. However, these studies fail to control for differences in health systems facilities and providers. By comparing patients hospitalized on a single medical service during the Delta and Omicron surges we were able to conduct a more accurate comparison of the two varaints’ clinical manifestations and outcomes.

**Methods:**

We conducted a prospective study of 364 Omicron (BA.1) infected patients on a single hospitalist service and compared these findings to a retrospective analysis of 241 Delta variant infected patients managed on the same service. We examined differences in symptoms, laboratory measures, and clinical severity between the two variants and assessed potential risk drivers for case mortality.

**Findings:**

Patients infected with Omicron were older and had more underlying medical conditions increasing their risk of death. Although they were less severely ill and required less supplemental oxygen and dexamethasone, in-hospital mortality was similar to Delta cases, 7.14% vs. 4.98% for Delta (q-value = 0.38). Patients older than 60 years or with immunocompromised conditions had much higher risk of death during hospitalization, with estimated odds ratios of 17.46 (95% CI: 5.05, 110.51) and 2.80 (1.03, 7.08) respectively. Neither vaccine history nor variant type played a significant role in case fatality. The Rothman score, NEWS-2 score, level of neutrophils, level of care, age, and creatinine level at admission were highly predictive of in-hospital death.

**Interpretation:**

In hospitalized patients, the Omicron variant is less virulent than the Delta variant but is associated with a comparable mortality. Clinical and laboratory features at admission are informative about the risk of death.

**Supplementary Information:**

The online version contains supplementary material available at 10.1186/s12879-023-08714-x.

## Introduction

The SARS-CoV-2 pandemic has consisted of multiple surges of infection because of continuous genetic and antigenic drifting that allowed immune-escape and the generation of more contagious variants, beginning with the Alpha variant, followed by the Beta variant and the Delta variant. In November of 2021 South Africa experienced a large surge due to a variant designated as the Omicron (BA1) variant that was estimated to be 2–3 times more contagious than the Delta variant [1]. However, the high incidence of infections in South Africa “was decoupled from the incidences of hospitalization, recorded death, and excess death.“ This decoupling was partially explained by the high seroprevalence of anti-SARS-COVID-2 IgG antibodies (56.2–79.7%) prior to the Omicron wave indicating significant prior exposure and immunity [2].

Subsequent epidemiology studies in multiple developed countries [3–14] suggest that the Omicron variant causes less severe disease and a lower mortality than previous variants. Analysis of hospitalized patients in South Africa revealed a lower incidence of severe disease and reduced mortality [15, 16].

New Omicron variants including BA2 and BA5 and more recently BQ1 and BQ1.1 contain mutations that circumvent immunity directed against the SARS-CoV-2 ancestral strain and the original vaccines, and consequently are now spreading throughout the world. To date these variants have not been shown to differ in clinical severity as compared to BA1 [17].

To directly compare the severity and differences in the clinical presentation of Omicron to the Delta variant we prospectively studied 362 patients admitted during the peak of the local Omicron surge in North Florida. We then collected data on a retrospective cohort of 241 randomly selected cases admitted to the same hospitalist service during the Delta variant surge. All patients were managed by the same physicians and nurses eliminating institutional differences in case selection and clinical resources allowing a more accurate comparison of virulence defined as the severity of disease manifestations as assessed by the clincal symptoms (fever, chills and shortness of breath); vital signs (respiratory rate, heart rate, blood pressure and room air oxygen saturation); laboratory findings (inflammatory markers, d-dimer and creatinine); imaging studies (chest Xray or chest CT scan); and mortality. Finally we have applied machine learning to create an accurate predictive model of fatal outcome to identify patients requiring early escalation of care.

## Methods

### Patient population

All patients were hospitalized on the Hospitalist Service at the University of Florida Shands Hospital, a major academic referral center for Northern Florida and Southern Georgia with 1,162 licensed beds. The Hospitalist Service consists of 3 Medical Surgical wards (36 beds each) and 2 Intermediate Care (IMC) units (30 and 24 beds). Throughout the study the same selection criteria for hospital admission of COVID-19 were applied to all patients. (Appendix, Supplementary Methods)

### Patient clinical data entry

**Fig. 1:**
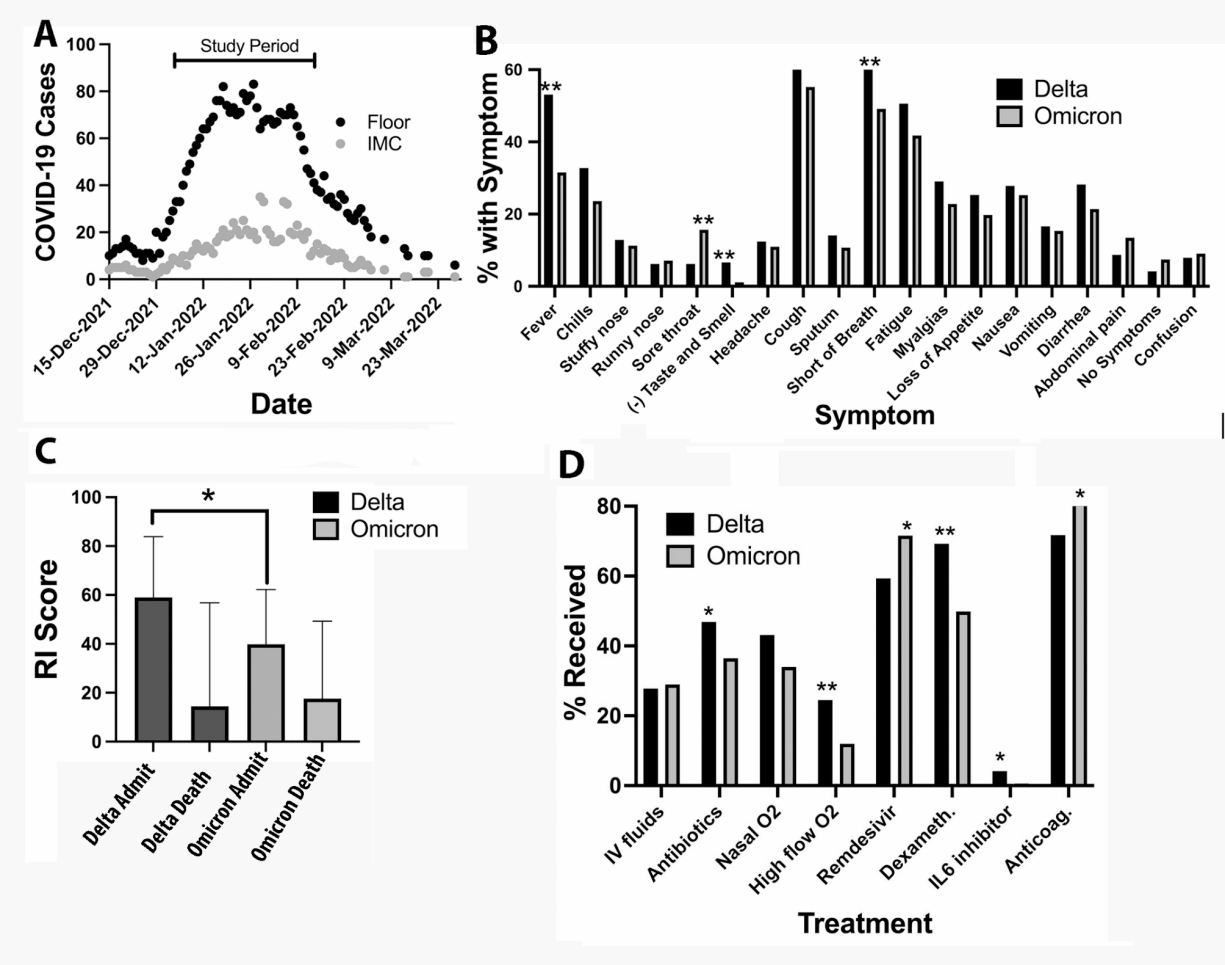
(**A**) Daily cases during the period of the prospective study of Omicron-infected patients Bracket marks the period when cases were studied. Genomic sequencing of 1 out of 3 patients during this time period revealed 99.2% of cases were the Omicron BA1 variant. (**B**) Percentage of Delta and Omicron-infected patients who had the reported symptoms associated with their infection. ** p < 0.001 (see Table 1 for statistical analysis). (**C**) Rothman index scores on admission and at the time of death for Omicron and Delta-infected patients who died in the hospital. Bracket marks the statistically significant difference in admission Rothman Index scores, Delta scores being higher (better overall health) than Omicron cases, Mean ± SD 58.2 ± 26.9 n = 8 vs. 34.8 ± 23.0 n = 34. *p = 0.0158. (**D**) Percentage of Delta and Omicron-infected patients who received each treatment * p = 0.039 − 0.009. ** p = < 0.001 (See Table 1 for statistical analysis)

The prospective Omicron study began on December 30, 2021 and continued through February 15, 2022 during the peak of COVID-19 hospital admissions (Fig. 1A). Patients with either (1) confirmation by genome sequencing or (2) a positive RTPCR and a symptom onset on or after Jan. 8, 2022 were consider to be infected with Omicron. This cut-off date was chosen according to the proportions of variants in the southeastern health region of the US (Appendix, Fig. S1).

Twelve hospitalists were assigned in rotation to review the charts of all patients admitted to the Hospitalist Service with a positive RTPCR test for SARS-COV-2 during the period from December 30th to February 15th. A RedCap data entry form with 64 possible data entries was filled out (Appendix, Fig. S4) https://www.project-redcap.org/software/ In addition to standard clinical parameters, we recorded 2 global measures of clinical status: NEWS-2 score and Rothman Index. NEWS-2, National Early Warning Score 2 includes respiratory rate, O2 saturation, temperature, blood pressure, heart rate, and level of consciousness. Scores range from 0 to 20 and the higher the score the higher the risk of a fatal outcome [18]. The Rothman Index incorporates 26 values including 10 nursing functional assessments, vital signs and basic laboratory values and has been verified in multiple studies [19, 20]. Scores range from 100 to -91, the lower the score the poorer the patient’s overall medical condition and the worse their prognosis. Illness severity was also classified as mild, moderate, severe or critical using the WHO criteria as of 12/2022 (Appendix, Supplemental Methods).

All patients were followed until hospital discharge or death. Patients who died within 30 days of discharge were also included. The cause of death was independently determined by two physicians (NR and FS) who after discussions achieved 100% agreement (Appendix Supplemental Methods). The reason for admission, COVID-19 or other, was determined by the individual reviewer. There was 87.5% agreement as to the cause of admission between 2 independent duplicate reviewers of 40 charts.

For the retrospective analysis of Delta variant patients records were randomly selecting from the Electronic Health Record data base from June 19, 2021 to October 14, 2021 using the search terms COVID-19 and MHS (hospitalist service). During this period genomic sequence analysis revealed that over 95% of all COVID-19 cases were due to the Delta variant in the U.S. [21] Three additional Delta cases were identified by genomic sequencing on January 3, 7, and 16, 2022.

### Statistical analysis

We summarized the demographic and clinical characteristics for all patients as well as for subgroups defined by vaccine status (vaccinated vs. unvaccinated) and variant type (Delta vs. Omicron). The overall research design could be classified as a retrospective cohort study. Categorical characteristics were compared between subgroups using the Fisher’s exact test, and continuous variables were compared using the Wilcoxon rank sum test. Multiple comparisons were adjusted to control the false discovery rate at 0.05 using the Benjamin-Hochberg procedure, and both the unadjusted p-values and multi-comparison-adjusted q-values were reported.

To assess the impact of vaccination and variant on survival, we fitted multivariable logistic models to the binary outcome of death, adjusting for demographic features and chronic conditions. Baseline clinical signs, symptoms, severity, treatment and management variables were not included as vaccines could change these variables. Models were generated for four definitions of endpoints: 1) all-cause in-hospital deaths, (2) all-cause deaths that occurred in-hospital or within 30 days of discharge,(3) COVID-attributed in-hospital deaths, (4) COVID-attributed deaths that occurred in-hospital or within 30 days of discharge; We regard the 1st endpoint as the most clinically relevant. We first screened variables using Fisher’s exact test for univariable association using the broadest definition of mortality (all-cause deaths in the hospital or within 30 days of discharge) as the most inclusive definition, and variables with p-values < 0.1 were retained for model building. We then fit the models combining all four endpoints. At each step, the smallest p-value across all four models was calculated for each variable, and the variable with the largest p-value was dropped. This backward selection procedure was stopped when all remaining variables had a smallest p-value < 0.05. We also conducted backward selection individually for each model to obtain parsimonious models, variables with p-values > 0.05 being sequentially dropped from each model.

To guide clinical management of patients with a high risk of death, we developed a prediction model for death using a machine learning algorithm, the Boosted Regression Trees (BRT), implemented in the XGBOOST R package. (Appendix, Supplemental Methods) All statistical analyses were conducted in the software R version 4.2.

## Results

A total of 614 SARS-CoV-2 infected patients admitted during the period from June 19, 2021 to February 15, 2022 were studied. Among these patients, 241 were considered to be infected with Delta, dates of positive RTPCR tests spanning June 19, 2021 to January 16, 2022, of whom 238 were admitted and tested on or before October 15, 2021 when Delta was the dominant strain (Appendix, Fig. S2). The remaining three were admitted in January of 2022 and identified by genome sequencing. The other 373 patients were admitted and tested after November 16, 2021, of whom 121 were confirmed to be Omicron by genome sequencing, and 252 were not sequenced. During the period of January 8 to February 15, 2022, nearly one third of patients (107/350) underwent full genomic sequencing, and only one patient admitted on January 16, 2022 was identified as a Delta variant. The other 106 sequences identified the Omicron BA.1 variant (99.1%), consistent with the composition of variants in the southeastern health region of the US (Appendix, Fig. S1). It is hence reasonable to consider the 243 unsequenced patients tested during this period as infected by Omicron. Our analyses were thus based on 241 Delta patients and 364 (121 + 243) Omicron patients.

Comparisons of demographics, clinical symptoms, baseline health conditions, baseline clinical measurements, clinical management, and survival between Delta-infected and Omicron-infected hospitalized patients revealed 29 out of 64 (45.3%) statistically meaningful differences with q-values ≤0.05 (Table 1). As compared to Delta, Omicron-infected patients were older (39.84% vs. 26.56% ≥70 years), were less likely to be admitted because of COVID-19 (58.24% vs. 74.69%), more likely to be vaccinate;d (37.36% ≥2 doses vs. 12.45%), and more likely to have chronic conditions such as cardiovascular disease (29.95% vs. 17.84%) and hypertension (57.42% vs. 42.32%).

**Table 1:** Characteristics of patients infected by the Omicron and Delta Variants*

Characteristics	Category	Delta	Omicron	*p*-value	*q*-value
Total		241 (100%)	364 (100%)		
Age Group	20–39 years old	43 (17.8)	41 (11.3)	0.001	**0.005**
	40–69 years old	134 (55.6)	178 (48.9)		
	70 years or older	64 (26.6)	145 (39.8)		
Gender	Female	118 (49.0)	202 (55.5)	0.134	0.195
Reason of Admission	COVID-19	180 (74.7)	212 (58.2)	< 0.001	**< 0.001**
Vaccination Status	None	200 (83.0)	208 (57.1)	< 0.001	**< 0.001**
	1 shot	11 (4.6)	20 (5.5)		
	2 shots	26 (10.8)	97 (26.6)		
	3 shots	4 (1.7)	39 (10.7)		
Symptoms	Fever	128 (53.1)	115 (31.59)	< 0.001	**< 0.001**
	Chills	79 (32.8)	86 (23.6)	0.015	**0.039**
	Headache	30 (12.4)	40 (11.0)	0.605	0.655
	Stuffy nose	31 (12.9)	41 (11.3)	0.608	0.655
	Runny nose	15 (6.2)	26 (7.1)	0.742	0.766
	Sore throat	15 (6.2)	57 (15.6)	< 0.001	**0.002**
	Loss of Taste and Smell	16 (6.6)	4 (1.1)	< 0.001	**0.002**
	Earache	1 (0.4)	0 (0.00)	0.398	0.472
	Cough	153 (63.5)	201 (55.2)	0.052	0.102
	Sputum production	34 (14.1)	39 (10.7)	0.251	0.321
	Shortness of Breath	148 (61.4)	179 (49.2)	0.004	**0.013**
	Fatigue	122 (50.6)	152 (41.8)	0.037	0.076
	Myalgia	70 (29.0)	83 (22.80	0.086	0.150
	Loss of Appetite	61 (25.3)	72 (19.8)	0.110	0.172
	Nausea	67 (27.8)	92 (25.3)	0.510	0.583
	Vomiting	40 (16.6)	56 (15.4)	0.733	0.766
	Diarrhea	68 (28.2)	78 (21.4)	0.065	0.119
	Abdominal pain	21 (8.7)	49 (13.5)	0.091	0.151
	No Symptoms	10 (4.2)	27 (7.4)	0.119	0.182
	Confusion, altered mental status	18 (7.5)	33 (9.1)	0.551	0.619
	Other	46 (19.1)	81 (22.2)	0.361	0.436
Baseline Diseases	Cardiovascular Disease	43 (17.8)	109 (30.0)	< 0.001	**0.004**
	Hypertension	102 (42.3)	209 (57.4)	< 0.001	**0.002**
	Diabetes Mellitus	58 (24.1)	111 (30.5)	0.096	0.153
	COPD	27 (11.2)	54 (14.8)	0.223	0.298
	Obesity	37 (15.4)	42 (11.5)	0.177	0.242
	Sickle Cell Disease	0 (0.00)	4 (1.1)	0.155	0.216
	Asthma	11 (4.6)	28 (7.7)	0.132	0.195
	Active Cancer	9 (3.7)	24 (6.6)	0.146	0.208
	Immunocompromised	17 (7.0)	41 (11.3)	0.092	0.151
	Other underlying disease	77 (32.0)	143 (39.3)	0.070	0.125
CXR or Chest CT	Clear	47 (19.5)	144 (39.6)	< 0.001	**< 0.001**
	Unilateral infiltrate	20 (8.3)	49 (13.5)		
	Multifocal infiltrates	169 (70.1)	157 (43.1)		
	ARDS (ext. pulmonary edema)	0 (0.0)	2 (0.6)		
Severity	Mild	45 (18.7)	131 (36,0)	< 0.001	**< 0.001**
	Moderate	46 (19.1)	74 (20.3)		
	Severe	119 (49.4)	143 (39.3)		
	Critical	31 (12.9)	16 (4.4)		
Level of Care	Medical/Surgical Floor	162 (67.2)	270 (74.2)	0.041	0.082
	IMC	55 (22.8)	76 (20.9)		
	MICU	24 (10.0)	18 (5.0)		
Days from Onset to Admission**		7 (3, 10)	3 (2, 7)	< 0.001	**< 0.001**
Days from PCR to Admission***		0 (0, 1)	0 (0, 0)	0.009	**0.027**
		Mean (95% CI).	Mean (95% CI)		
Baseline Laboratory	O2 Saturation RA	92.0%(88.0, 95.0)	94.0%(90.0, 96.0)	< 0.001	**0.001**
Measurements	Respiratory Rate	22.0/min (18.0, 28.0)	20.0 (18.0, 25.0)	0.012	**0.033**
	NEWS-2 Score	5.0 (3.0, 7.0)	4.0 (2.0, 7.0)	0.036	0.076
	WBC	6200/µl (4400, 9200)	6900/µl (5000, 9800)	0.065	0.119
	Neutrophils	77.7% (68.9, 84.0)	74.6% (64.20, 83.0)	0.009	**0.026**
	Lymphocytes	750/µl (500, 1175)	880/µl (600, 1425)	0.007	**0.025**
	Creatinine	0.96 mg/dL (0.77, 1.27)	1.01 mg/dL (0.79, 1.45)	0.239	0.312
	CRP	70.0 mg/dL(30.6, 135.5)	42.97 (12.89, 121.16)	< 0.001	**0.003**
	Procalcitonin	0.10 ng/mL (0.06, 0.24)	0.10 ng/mL (0.05, 0.28)	0.614	0.655
	Baseline Rothman Score	73.0 (59.0, 83.0)	70.0 (51.0, 81.0)	0.012	**0.033**
	D dimer	1.00 mg/L (0.58, 1.98)	1.08 (0.60, 1.84)	0.832	0.845
Management	IV fluids	67 (27.8)	104 (28.6)	0.854	0.854
	Antibiotics	113 (46.9)	136 (37.4)	0.023	**0.050**
	Nasal O2	104 (43.2)	122 (33.5)	0.020	**0.048**
	High flow O2	59 (24.5)	45 (12.4)	< 0.001	**0.001**
	Remdesivir	143 (59.3)	260 (71.4)	0.003	**0.011**
	Dexamethasone	167 (69.3)	181 (49.7)	< 0.001	**< 0.001**
	IL6 inhibitor	10 (4.2)	3 (0.8)	0.008	**0.026**
	Other	17 (7.0)	48 (13.2)	0.022	**0.050**
	Anticoagulation	173 (71.8)	297 (81.6)	0.005	**0.019**
Death (All Cause)	In-hospital	12 (5.0)	26 (7.1)	0.309	0.381
	In-hospital or ≤30 days of discharge	15 (6.2)	45 (12.4)	0.018	**0.043**
Death (COVID-19)	In-hospital	10 (4.2)	21 (5.8)	0.453	0.527
	In-hospital or ≥of discharge	12 (5.0)	26 (7.1)	0.309	0.381

* Frequency (percent) for qualitative and median (IQR) for quantitative characteristics,. q-values≤0.05 (bolded) control the false discovery rate to be ≤5%

** Interval from first symptom to the time of admission

*** Interval from the time the RTPCR was positive to the time of admission

As shown in Table 1; Fig. 1B, among major symptoms (prevalence ≥20%) the two variants caused similar frequencies of cough, myalgia, loss of appetite, nausea, and diarrhea. The Omicron patients were less likely to present with fever (31.59% vs. 53.11%), chills (23.63% vs. 32.78%), shortness of breath (49.18% vs. 61.41%), and loss of taste and smell (1.1% vs. 6.64%). The only symptom more likely in Omicron patients with a q-value < 0.05 was sore throat (15.66% vs. 6.22%).

Respiratory rate, oxygenation, and NEWS-2 scores at admission were less severely affected in Omicron patients. Omicron patients had slightly lower Rothman index (median [IQR] = 70 vs. 73). The gap in admission Rothman index was larger among fatal cases, nearly 24 points lower for Omicron (Mean 58.25 vs. 34.79, p = 0.0158, Fig. 1C) indicating poorer overall health. Laboratory findings demonstrated a lower degree of inflammation as assessed by neutrophil response (74.6 vs. 77.7) (for confidence intervals see Table 1) and C-reactive protein (42.97 vs. 70.0) and the reduction in total lymphocyte count was less severe in Omicron variant patients (880 vs. 750). Omicron-infected patients were more likely to have a clear chest X-ray or CT scan (39.56% vs. 19.5%) and the overall rating of the severity of their infection was lower as compared to Delta patients (43.69% vs. 62.24% severe or critical). The median delay from symptom onset to admission was 3 days for Omicron patients, much shorter than 7 days for Delta patients.

As shown in Fig. 1D fewer Omicron patients were prescribed antibiotics (37.36% vs. 46.89%), high flow oxygen (12.36% vs. 24.48%), Dexamethasone (49.73% vs. 69.29%) and IL6 inhibitors (0.82% vs. 4.15%). However, a significantly higher number were given Remdesivir (71.43% vs. 59.34%) and anticoagulated (81.59% vs. 71.78%), reflecting a systematic change in clinical management. (see discussion).

To eliminate potential confounding effects of vaccination on the clinical manifestations, baseline laboratory measurements, and clinical management and outcome, we restricted comparisons to unvaccinated hospitalized patients and identified similar statistically significant differences. (Appendix, Table S1).

Despite the reduced severity of COVID-19 in hospitalized patients infected with the Omicron variant, all-cause mortality during hospitalization or within 30 days of hospital discharge was higher in Omicron compared to Delta infected patients (12.36% vs. 6.22%, q = 0.043). However, this difference did not achieve statistical significance for all cause in-hospital mortality (7.14% vs. 4.98% q = 0.381), COVID-19 attributable mortality in-hospital (5.77% vs. 4.15%), or COVID-19 attributable mortality in-hospital or within 30 days of discharge (7.14% vs. 4.98%). A higher percentage of hospitalized Omicron patients died outside the hospital within 30 days of discharge (42.22% vs. 20%); however this difference did not achieve statistical significance (p = 0.215).

Multivariable regression analyses of the drivers for the risk of death among hospitalized Delta and Omicron patients are shown in Table 2. For all cause in-hospital death an elevated risk was associated with an age older than 60 years (OR = 17.46) (95% CI, see Table 2) immunocompromised condition (OR = 2.80), diabetes mellitus (OR = 2.15) and positive RT-PCR test before admission (OR = 2.17). A reduced risk of death was associated with chronic hypertension (OR = 0.39). Neither vaccine nor viral variant played a significant role in the risk of death among hospitalized patients. When deaths were expanded to include deaths within 30 days of discharge, the conclusions were comparable, but somewhat less significant. When the endpoint was restricted to deaths attributed to COVID-19, only age group and hypertension remained statistically significant with similar odds ratios. Admission unrelated to COVID-19 was associated with up to 5-fold lower risk of COVID-19-attributed death, with odds ratios of 0.14 for in-hospital death and 0.31 for deaths in the hospital and within 30 days of discharge. We performed a sensitivity analysis by resctricting Omicron patients to the 121 individuals confirmed by full genomic sequencing. This restriction was not applied to Delta patients as full genomic sequences were not started until the end of December of 2021. The results are qualitatively similar, although the age effects differ in magnitude, e.g., the odds ratio for age group regarding all-cause in-hospital death decreased from 17.46 to 9.75 (Appendix, Table S2).

**Table 2:** Logistic regression analysis of deaths among hospitalized patients infected by the Omicron and Delta Variants. Showing Odds Ratios^†^ (95% confidence intervals)

Variables	COVID-attributed Deaths		All-Cause Deaths	
	In-hospital	In-hospital + ≤ 30 days of discharge	In-hospital	In-hospital + ≤ 30 days of discharge
Age group ( > = 60 vs. <60)	**13.10 (3.71, 83.60)**	**8.04 (3.06, 27.75)**	**17.46 (5.05, 110.51)**	**9.66 (4.06, 28.70)**
Immunocompromised (Yes vs. No)	2.58 (0.87, 7.02)	1.90 (0.68, 4.77)	**2.80 (1.03, 7.08)**	2.26 (0.95, 5.06)
Diabetes Mellitus (Yes vs. No)	1.77 (0.74, 4.09)	1.36 (0.64, 2.81)	**2.15 (1.01, 4.51)**	1.67 (0.90, 3.04)
Hypertension (Yes vs. No)	**0.37 (0.16, 0.83)**	**0.42 (0.21, 0.85)**	**0.39 (0.18, 0.80)**	0.61 (0.34, 1.09)
Other underlying diseases (Yes vs. No)	1.32 (0.59, 2.90)	1.43 (0.72, 2.80)	1.32 (0.64, 2.67)	**1.77 (1.0, 3.13)**
Reason of admission (Other vs. COVID-19)	**0.14 (0.022, 0.48)**	**0.31 (0.11, 0.71)**	0.67 (0.29, 1.47)	0.98 (0.53, 1.78)
Positive RT-PCR test before admission^‡^ (Yes vs. No)	2.04 (0.91, 4.50)	1.46 (0.70, 2.95)	**2.17 (1.03, 4.51)**	1.65 (0.87, 3.06)
Variant (Delta vs. Omicron)	0.82 (0.34, 1.87)	0.67 (0.31, 1.39)	0.86 (0.39, 1.83)	0.59 (0.30, 1.10)
Vaccinated (≥1 vs. 0 doses)	1.38 (0.60, 3.14)	1.21 (0.59, 2.45)	1.33 (0.63, 2.78)	1.09 (0.60, 1.96)

† Odds Ratios stratified by whether death was attributed to COVID-19 and whether death occurred during hospitalization. Results with statistical significance are bolded

‡ Positive RT-PCR test before admission vs. after admission correspond to days from RT-PCR + to admission > 0 vs. ≤0

When the logistic models were solely applied to Omicron patients (Table 3), similar estimates were obtained; however, the OR estimates increased from 17.46 to 20.08 for age group and from 2.80 to 4.17 for immunocompromised condition when we restricted the analysis to all-cause in-hospital deaths. These higher OR estimates suggest that age and immuncompromised conditions are more influential for Omicron patients compared to Delta patients.

**Table 3:** Logistic regression of death outcome among hospitalized patients infected with Omicron. Odds ratios^†^ (95% confidence intervals)

Variables	COVID-attributed Deaths		All-Cause Deaths	
	In-hospital	In-hospital + ≤ 30 days of discharge	In-hospital	In-hospital + ≤ 30 days of discharge
Age group ( > = 60 vs. <60)	**14.79 (2.85, 273.54)**	**9.73 (2.74, 62.28)**	**20.07 (3.97, 367.73)**	**11.26 (3.82, 48.59)**
Immunocompromised (Yes vs. No)	**4.01 (1.18, 12.87)**	2.57 (0.82, 7.31)	**4.17 (1.37, 11.98)**	**3.13 (1.21, 7.76)**
Hypertension (Yes vs. No)	0.39 (0.15, 1.01)	**0.42 (0.19, 0.95)**	**0.42 (0.18, 0.99)**	0.66 (0.34, 1.30)
Other underlying diseases (Yes vs. No)	1.81 (0.69, 4.83)	1.71 (0.76, 3.85)	1.96 (0.84, 4.73)	**2.45 (1.26, 4.85)**
Reason of admission (Other vs. COVID-19)	**0.16 (0.025, 0.58)**	**0.39 (0.14, 0.96)**	0.61 (0.22, 1.49)	1.11 (0.56, 2.18)
Vaccinated (≥1 vs. 0 doses)	1.51 (0.56, 4.13)	1.30 (0.57, 2.97)	1.55 (0.65, 3.79)	1.19 (0.60, 2.33)

† Results are stratified by whether death was attributed to COVID-19 and whether death occurred during hospitalization. Results with statistical significance are bolded

To obtain models that were as parsimonious as possible, we also conducted backward selection for each endpoint separately. Results in Tables S3 and S4 in the appendix closely resembled those for the corresponding variables and models in Tables 2 and 3.

Using the XGBOOST algorithm, we identified initial Rothman score and neutrophils as the two most influential predictors for the risk of death regardless of the endpoint definition. (Table 4) We used a traditional cut-off of 5 for the importance score, i.e., an importance score ≥5 was considered an important predictor. The importance scores ranged 15.88–39.66 for initial Rothman score and 9.11–10.86 for neutrophils across the four endpoints. Creatinine, age group and CRP were also important predictors for three of four endpoints. NEWS2 score, he level of care, days from positive RTPCR to admission, and O_2_ saturation were important for two endpoints. When restricted to all cause in-hospital death, all eight factors mentioned above except for O_2_ saturation were important. The predictive performance was satisfactory for all endpoint definitions, with the areas under curve (AUC) reaching 95.74–98.81% for the training sets and 78.45–85.47% for the testing sets. We plotted the average response curves for the important predictors for all cause in-hospital death (appendix, Fig. S2) demonstrating a higher risk of death in association with admission to the MICU, an older age (> 70 years), a lower initial Rothman score (≤40), and higher levels of NEWS2 score (≥10), creatinine ((≥2.7) and neutrophils (≥90%).

**Table 4:** Machine learning algorithm identifying the importance predictive performance (%) for death in-hospitalized patients infected with Delta or Omicron*

	Variable or Dataset	COVID-attributed Deaths		All-Cause Deaths	
		In-hospital	In-hospital + ≤ 30 days of discharge	In-hospital	In-hospital + ≤ 30 days of discharge
Importance	Initial Rothman Score	**16.41**	**39.66**	**15.88**	**36.35**
	News-2 Score	**12.56**	4.88	**13.60**	3.90
	Neutrophils	**9.11**	**9.96**	**10.86**	**10.61**
	Level of Care	**7.73**	3.19	**9.47**	3.88
	Days from PCR + to Adm.	**6.51**	3.41	**5.62**	3.08
	O_2_ Saturation	**5.31**	**7.31**	4.19	4.27
	Creatinine	**6.81**	3.15	**7.97**	**5.95**
	Age Group	**5.63**	4.99	**9.51**	**6.41**
	Respiratory Rate	4.44	2.39	4.47	4.84
	CRP	**8.08**	**6.12**	**5.08**	4.88
	Lymphocytes	3.44		3.11	**5.43**
	WBC	4.34	**6.08**	4.25	4.33
	Procalcitonin	3.88	4.90	3.49	3.84
	Initial Severity	2.62		2.49	
	Days from symptom onset to PCR+	3.11	3.95		
	Confusion/altered mental status				2.22
AUC	Training	97.1	95.7	98.8	96.8
	Testing	83.8	81.7	85.5	78.4

*based on the XGBOOST algorithm. Importance scores ≥5 are bolded

## Discussion

The assessment of intrinsic virulence of different SARS-CoV-2 variants is critical for determining future public health needs for hospital resources and for informing the managing clinicians’ expectations for hospital course and therapeutic needs. Studies on the intrinsic virulence of the Omicron variant in hamsters and human ACE-2 expressing mice revealed milder disease manifestations and reduced inflammation in the naso-olfactory region and lungs as compared to infection with the Delta variant [22–25].

However, animal studies are difficult to extrapolate to humans. Population-level studies are fraught with potential confounding issues and must be interpreted with caution, particularly when it comes to the Omicron variant because of the marked variability of immunity in different populations [26]. The marked dissociation between infection rate and hospitalization and death rates in South Africa has been attributed to the high level of immunity within the population rather than differences in virulence [2, 15, 27, 28]. Additional population studies from other countries including Canada [3], Denmark [4], France [5], Germany [6], Sweden [7], United Kingdom [8–10] and the United States [11–14], have confirmed the lower hospitalization and death rates for Omicron versus earlier variants, particularly the Delta variant, but these studies have been cautious with regards to inferring reduced intrinsic virulence.

Hospital-based clinical series provide more direct evidence. In addition to the Los Angeles study reported early in the U.S. Omicron surge that reported a reduced mortality (4% versus 8.3% for Delta) [29] a series of infected pregnant women from Parkland Health in Dallas revealed a much lower percentage of severe disease among the women infected with Omicron as compared with those infected with Delta, (0.9% vs. 11.8%,) [30]. A comparison of patients with genotype proven Omicron (n = 274) and Delta (n = 35) hospitalized at Stanford University Hospital in California revealed less severe disease among Omicron patients (23.1% vs. 57.0%); however, the number of Delta cases was small [31]. Recent studies of hospitalized patients in Belgium and Norway also found Omicron-infected patients had lower likelihood of placement in the MICU [32, 33], and both studies found lower mortality rates among Omicron-infected hospitalized patients; however, this difference did not achieve statistical significance in the Belgium study [33].

Large studies that include multiple hospitals or multiple clinical services fail to control for differences in staff expertise, admission criteria, and capacity for timely treatment. A strength of our study was the ability to achieve maximal control of these variables. Both Omicron and Delta patients were admitted and treated on the same university hospitalist service. Our study supports previous conclusions that Omicron infection results in less severe clinical manifestations as compared to the Delta variant. We identified 9 statistically significant differences consistent with milder disease: oxygen saturation at room air, respiratory rate, NEWS2 score, neutrophil count, lymphocyte count, C-reactive protein, CXR infiltrates, WHO illness severity score and assigned level of care. (Table 1). This reduction in clinical severity was accompanied by a reduced need for high flow oxygen supplementation and anti-inflammatory treatment with corticosteroids and IL6 inhibitors.

With regards to clinical differentiation of Omicron and Delta based on symptoms, our findings matched those of an extensive United Kingdom survey of 4,990 Delta and 4,990 Omicron patients using a smart phone app. As observed in this study we found extensive overlap of symptoms with only fever, chills, loss of taste and smell, and shortness of breath being more prevalent in Delta patients and sore throat being more common among Omicron patients [34]. This close agreement suggests our patient population accurately reflected the clinical characteristics of COVID-19 patients in developed countries.

Limitations of our study include the modest number of cases and investigation of patients from a single geographic location, Northern Florida, a region that has an older population (21% ≥65 years) as compared to the overall U.S population (17%), but comparable to many European countries (19–24%). Secondly, the variant type was not confirmed by full genomic sequencing for most patients. Misclassification between Alpha and Delta before mid-July of 2021 was possible, but the Alpha variant has been found to have nearly comparable virulence to Delta and slight contamination would not be expected to compromise the comparisons. Misclassification between Delta and Omicron after January 8th, 2022 was highly unlikely as shown by both public virological surveillance (Appendix, Fig. S1) and our own sequence samples (Appendix, Fig. S2). In addition, the sensitivity analysis restricted to Omicron patients confirmed by genome sequencing showed qualitatively similar results. Thirdly, our study included only the BA1 Omicron variant, and it is possible that other Omicron variants may be associated with changes in virulence. Fourthly Omicron infected patients were hospitalized earlier in their course of illness possibly as a consequence of the repeated public health messages encouraging early medical evaluation. The earlier presentation of Omicron patients could in part explain the milder clinical manifestations of Omicron. However, in comparing changes in the Rothman Index over the duration of hospitalization there were no significant differences between the two variants (mean change in RI from admission to discharge, Omicron – 1.9, Delta − 1.2) indicating that Omicron patients’ clinical condition did not show greater deterioration than Delta patients during hospitalization. Finally, the Division of Hospital Medicine made two major changes in management protocols before the Omicron surge. Given the wide availability of Remdesivir we administered this medication within the first 12 h of admission for all symptomatic individuals. In addition, we emphasized the importance of ordering prophylactic anticoagulation for all patients hospitalized with COVID-19. These conditions would be expected to improve outcome and cannot explain our higher mortality rate as compared to previous Omicron studies.

A major difference between Delta and Omicron patients in our study and in previous studies was the level of vaccination. In the present study, Omicron patients had a much higher vaccination rate as compared to Delta patients (37.36% vs. 12.45%.) as expected. During 2022, more people would have been offered and taken the vaccines. This difference could explain the less severe clinical manifestations of patients infected with the Omicron variant. To avoid this potential confounder, we conducted a sensitivity analysis restricted to patients who had not been vaccinated and found similar differences in clinical manifestations between the two variants, indicating that prior immunity alone could not explain the less severe clinical manifestations of the Omicron variant and strongly suggesting that this variant has lower intrinsic virulence as compared to the Delta variant. Furthermore, logistic regression analysis revealed that vaccination was not a predictor of survival in our hospitalized Omicron patients (Tables 2 and 3). Our findings seemingly contradict a recent study in Finland reporting high vaccine efficacy in elderly patients against Omicron-associated hospitalization and ICU admission; [35] However, we assessed a different measure of vaccine efficacy prevention of death in hospitalized Omicron infected patients.

Finally, we created a predictive model for case fatality among hospitalized patients using a machine-learning approach (Table 4). The Rothman index score, NEWS2 score, the percentage of neutrophils in the peripheral smear, and age were identified as important predictors, while vaccination status and variant were not. This model achieved a high level of accuracy for both training (AUC 96–99) and testing (AUC 84 to 85) for predicting in-hospital mortality, providing a helpful prognostic tool for clinicians who may take early interventions to avert potentially fatal outcomes. Our model is in agreement with recent publications documenting the value of both the NEWS2 score (AUC 74) [36] and Rothman Index (AUC 81 to 84) [37, 38] for predicting in-hospital mortality in COVID-19 infection.

Despite the decreased virulence of Omicron we found this variant resulted in mortality rates comparable to the Delta variant. This seemingly contradictory finding can be explained by the debilitated state of our hospitalized Omicron patients. As compared to hospitalized Delta patients, Omicron patients were older (39.84% vs. 26.56% > 70 years of age) and were more likely to have underlying cardiovascular disease (29.95% vs. 17.84%). Among fatal COVID-19 patients the overall medical condition at the time of admission for Omicron infected patients was significantly lower than for those who died from the Delta variant (Rothman Index 58.25 ± 26.87 vs. 34.79 ± 22.96, Fig. 1C). This score objectively documented the weakened medical condition of fatal Omicron-infected patients at the time of admission and warned of a poor prognosis [37, 38].

In conclusion the comparison of Delta versus Omicron variant infected patients on the same clinical service confirms the lower virulence or clinical severity of the Omicron variant. However, the less virulent Omicron variant demonstrated higher levels of person to person spread resulting in a higher percentage of elderly, debilitated and immunocompromised being admitted to our hospital with SARS-CoV-2 infections. As a consequence we found that the in-hospital mortality rates for Omicron variant were comparable to Delta variant infection. These findings emphasize the importance of continued surveillance and infection control prevention measures for reducing mortality.

### Electronic supplementary material

Below is the link to the electronic supplementary material.

Supplementary Material 1

## Data Availability

The datasets used and/or analysed during the current study are available from the corresponding author on reasonable request.

## References

[1] Ito K, Piantham C, Nishiura H. Relative instantaneous reproduction number of Omicron SARS-CoV-2 variant with respect to the Delta variant in Denmark. J Med Virol 2021. 10.1002/jmv.27560 PMC9015237 34967453

[2] Madhi SA, Kwatra G, Myers JE et al. Population Immunity and Covid-19 severity with Omicron variant in South Africa. N Engl J Med 2022. 10.1056/NEJMoa2119658 PMC8908853 35196424

[3] Ulloa AC, Buchan SA, Daneman N, Brown KA (2022). Estimates of SARS-CoV-2 Omicron variant severity in Ontario, Canada. JAMA.

[4] Bager P, Wohlfahrt J, Bhatt S (2022). Risk of hospitalisation associated with Infection with SARS-CoV-2 Omicron variant versus Delta variant in Denmark: an observational cohort study. Lancet Infect Dis.

[5] Auvigne V, Vaux S, Strat YL (2022). Severe hospital events following symptomatic Infection with Sars-CoV-2 Omicron and Delta variants in France, December 2021-January 2022: a retrospective, population-based, matched cohort study. EClinicalMedicine.

[6] Sievers C, Zacher B, Ullrich A et al. SARS-CoV-2 omicron variants BA.1 and BA.2 both show similarly reduced Disease severity of COVID-19 compared to Delta, Germany, 2021 to 2022. Euro Surveill 2022; 27. 10.2807/1560-7917.ES.2022.27.22.2200396 PMC9164675 35656831

[7] Kahn F, Bonander C, Moghaddassi M et al. Risk of severe COVID-19 from the Delta and Omicron variants in relation to vaccination status, sex, age and comorbidities - surveillance results from southern Sweden, July 2021 to January 2022. Euro Surveill 2022; 27. 10.2807/1560-7917.ES.2022.27.9.2200121 PMC8895467 35241215

[8] Nyberg T, Ferguson NM, Nash SG (2022). Comparative analysis of the risks of hospitalisation and death associated with SARS-CoV-2 Omicron (B.1.1.529) and Delta (B.1.617.2) variants in England: a cohort study. Lancet.

[9] Sheikh A, Kerr S, Woolhouse M, McMenamin J, Robertson C, Collaborators EI. Severity of Omicron variant of concern and effectiveness of vaccine boosters against symptomatic Disease in Scotland (EAVE II): a national cohort study with nested test-negative design. Lancet Infect Dis; 2022. 10.1016/S1473-3099(22)00141-4 PMC9033213 35468332

[10] Krutikov M, Stirrup O, Nacer-Laidi H (2022). Outcomes of SARS-CoV-2 Omicron Infection in residents of long-term care facilities in England (VIVALDI): a prospective, cohort study. Lancet Healthy Longev.

[11] Lauring AS, Tenforde MW, Chappell JD (2022). Clinical severity of, and effectiveness of mRNA vaccines against, covid-19 from Omicron, Delta, and alpha SARS-CoV-2 variants in the United States: prospective observational study. BMJ.

[12] Paredes MI, Lunn SM, Famulare M, et al. Associations between SARS-CoV-2 variants and risk of COVID-19 hospitalization among confirmed cases in Washington State: a retrospective cohort study. Clin Infect Dis; 2022. 10.1093/cid/ciac279 PMC9047245 35412591

[13] Lewnard JA, Hong VX, Patel MM, Kahn R, Lipsitch M, Tartof SY. Clinical outcomes associated with SARS-CoV-2 Omicron (B.1.1.529) variant and BA.1/BA.1.1 or BA.2 subvariant Infection in southern California. Nat Med 2022. 10.1038/s41591-022-01887-z PMC10208005 35675841

[14] Skarbinski J, Wood MS, Chervo TC (2022). Risk of severe clinical outcomes among persons with SARS-CoV-2 Infection with differing levels of vaccination during widespread Omicron (B.1.1.529) and Delta (B.1.617.2) variant circulation in Northern California: a retrospective cohort study. Lancet Reg Health Am.

[15] Wolter N, Jassat W, Walaza S (2022). Early assessment of the clinical severity of the SARS-CoV-2 Omicron variant in South Africa: a data linkage study. Lancet.

[16] Maslo C, Friedland R, Toubkin M, Laubscher A, Akaloo T, Kama B (2022). Characteristics and outcomes of hospitalized patients in South Africa during the COVID-19 Omicron Wave compared with previous waves. JAMA.

[17] Shaheen N, Mohamed A, Attalla A (2022). Could the New BA.2.75 Sub-variant cause the emergence of a global epidemic of COVID-19? A scoping review. Infect Drug Resist.

[18] Engebretsen S, Bogstrand ST, Jacobsen D, Vitelli V, Rimstad R (2020). NEWS2 versus a single-parameter system to identify critically ill medical patients in the emergency department. Resusc Plus.

[19] Alarhayem AQ, Muir MT, Jenkins DJ (2019). Application of electronic medical record-derived analytics in critical care: Rothman Index predicts mortality and readmissions in surgical intensive care unit patients. J Trauma Acute Care Surg.

[20] Chan AS, Rout A, Adamo CRD, Lev I, Yu A, Miller K (2022). Palliative referrals in Advanced Cancer patients: utilizing the supportive and Palliative Care indicators Tool and Rothman Index. Am J Hosp Palliat Care.

[21] Lambrou AS, Shirk P, Steele MK (2022). Genomic surveillance for SARS-CoV-2 variants: predominance of the Delta (B.1.617.2) and Omicron (B.1.1.529) variants - United States, June 2021-January 2022. MMWR Morb Mortal Wkly Rep.

[22] Armando F, Beythien G, Kaiser FK (2022). SARS-CoV-2 Omicron variant causes mild pathology in the upper and lower respiratory tract of hamsters. Nat Commun.

[23] Mohandas S, Yadav PD, Sapkal G (2022). Pathogenicity of SARS-CoV-2 Omicron (R346K) variant in Syrian hamsters and its cross-neutralization with different variants of concern. EBioMedicine.

[24] Natekar JP, Pathak H, Stone S et al. Differential Pathogenesis of SARS-CoV-2 variants of concern in human ACE2-Expressing mice. Viruses 2022; 14. 10.3390/v14061139 PMC9231291 35746611

[25] Tarres-Freixas F, Trinite B, Pons-Grifols A (2022). Heterogeneous infectivity and Pathogenesis of SARS-CoV-2 variants Beta, Delta and Omicron in Transgenic K18-hACE2 and wildtype mice. Front Microbiol.

[26] Bhattacharyya RP, Hanage WP (2022). Challenges in inferring intrinsic severity of the SARS-CoV-2 Omicron variant. N Engl J Med.

[27] Jassat W, Abdool Karim SS, Mudara C, et al. Clinical severity of COVID-19 in patients admitted to hospital during the Omicron wave in South Africa: a retrospective observational study. Lancet Glob Health; 2022. 10.1016/S2214-109X(22)00114-0 PMC9116895 35597249

[28] Davies MA, Kassanjee R, Rousseau P (2022). Outcomes of laboratory-confirmed SARS-CoV-2 Infection in the Omicron-driven fourth wave compared with previous waves in the Western Cape Province, South Africa. Trop Med Int Health.

[29] Modes ME, Directo MP, Melgar M (2022). Clinical characteristics and outcomes among adults hospitalized with laboratory-confirmed SARS-CoV-2 Infection during periods of B.1.617.2 (Delta) and B.1.1.529 (omicron) variant predominance - one hospital, California, July 15-September 23, 2021, and December 21, 2021-January 27, 2022. MMWR Morb Mortal Wkly Rep.

[30] Adhikari EH, MacDonald L, SoRelle JA, Morse J, Pruszynski J, Spong CY (2022). COVID-19 cases and Disease severity in pregnancy and neonatal positivity Associated With Delta (B.1.617.2) and Omicron (B.1.1.529) variant predominance. JAMA.

[31] Ozdalga E, Ahuja N, Sehgal N et al. Detailed characterization of hospitalized patients infected with the Omicron variant of SARS-CoV-2. J Intern Med 2022. 10.1111/joim.13501 PMC9115094 35417053

[32] Stalcrantz J, Kristoffersen AB, Boas H et al. Milder Disease trajectory among COVID-19 patients hospitalised with the SARS-CoV-2 Omicron variant compared with the Delta variant in Norway. Scand J Public Health 2022:14034948221108548. 10.1177/14034948221108548 35799474

[33] Van Goethem N, Chung PYJ, Meurisse M et al. Clinical severity of SARS-CoV-2 omicron variant compared with Delta among hospitalized COVID-19 patients in Belgium during autumn and winter season 2021–2022. Viruses 2022; 14. 10.3390/v14061297 PMC9227815 35746768

[34] Menni C, Valdes AM, Freidin MB (2020). Real-time tracking of self-reported symptoms to predict potential COVID-19. Nat Med.

[35] Baum U, Poukka E, Leino T, Kilpi T, Nohynek H, Palmu AA (2022). High vaccine effectiveness against severe COVID-19 in the elderly in Finland before and after the emergence of Omicron. BMC Infect Dis.

[36] Veldhuis L, Ridderikhof ML, Schinkel M (2021). Early warning scores to assess the probability of critical Illness in patients with COVID-19. Emerg Med J.

[37] Moguillansky D, Sharaf OM, Jin P (2022). Evaluation of clinical predictors for major outcomes in patients hospitalized with COVID-19: the potential role of the Rothman Index. Cureus.

[38] Beals Jt, Barnes JJ, Durand DJ (2021). Stratifying deterioration risk by acuity at admission offers triage insights for Coronavirus Disease 2019 patients. Crit Care Explor.

