# Correction: Comparison of the clinical characteristics of SARS-CoV-2 Delta (B.1.617.2) and Omicron (B.1.1.529) infected patients from a single hospitalist service

**DOI:** 10.1186/s12879-023-08827-3

**Published:** 2023-12-12

**Authors:** N. Radhakrishnan, M. Liu, B. Idowu, A. Bansari, K. Rathi, S. Magar, L. Mundhra, J. Sarmiento, U. Ghaffar, J. Kattan, R. Jones, J. George, Y. Yang, F. Southwick

**Affiliations:** 1 Division of Hospital Medicine, Department of Medicine, University of Florida College of Medicine, 6362 NW 41St Ave, Gainesville, FL 32606 USA; 2 Department of Biostatistics, College of Public Health and Health Professions, University of Florida, Gainesville, FL USA; 3 Department of Statistics, Franklin College of Arts and Sciences, University of Georgia, 310 Herty Drive, 30602 Athens, GA Greece

**BMC Infectious Diseases 23, 747 (2023)**

**https://doi.org/10.1186/s12879-023-08714-x**

The original publication of this article contained an incorrect caption for Fig. 1. The incorrect and correct information is listed in this correction article. The original article has been updated.

Incorrect

 (A) Daily cases during the period of the prospective study of Omicron-infected patients Bracket marks the period when cases were studied. Genomic sequencing of 1 out of 3 patients during this time period revealed 99.2% of cases were the Omicron BA1 variant. (B) Percentage of Delta and Omicron-infected patients who had the reported symptoms associated with their infection. ** p < 0.001 (see Table 1 for statistical analysis). (C) Percentage of Delta and Omicron-infected patients who received each treatment * p = 0.039 − 0.009. ** p =  < 0.001 (See Table 1 for statistical analysis). (D) Rothman index scores on admission and at the time of death for Omicron and Delta-infected patients who died in the hospital. Bracket marks the statistically significant difference in admission Rothman Index scores, Delta scores being higher (better overall health) than Omicron cases, Mean ± SD 58.2 ± 26.9 n = 8 vs. 34.8 ± 23.0 n = 34. *p = 0.0158.

Correct

(A) Daily cases during the period of the prospective study of Omicron-infected patients Bracket marks the period when cases were studied. Genomic sequencing of 1 out of 3 patients during this time period revealed 99.2% of cases were the Omicron BA1 variant. (B) Percentage of Delta and Omicron-infected patients who had the reported symptoms associated with their infection. ** p < 0.001 (see Table 1 for statistical analysis). (C) Rothman index scores on admission and at the time of death for Omicron and Delta-infected patients who died in the hospital. Bracket marks the statistically significant difference in admission Rothman Index scores, Delta scores being higher (better overall health) than Omicron cases, Mean ± SD 58.2 ± 26.9 n = 8 vs. 34.8 ± 23.0 n = 34. *p = 0.0158(D) Percentage of Delta and Omicron-infected patients who received each treatment * p = 0.039 − 0.009. ** p =  < 0.001 (See Table 1 for statistical analysis).
