# Supplementary material for: Comparison of the clinical characteristics of SARS-CoV-2 Delta (B.1.617.2) and Omicron (B.1.1.529) infected patients from a single hospitalist service
Source: BMC Infect Dis. 2023 Oct 31;23:747. doi: 10.1186/s12879-023-08714-x (PMC10617227; doi:10.1186/s12879-023-08714-x)
Supplement: Supplementary file 1 — Supplementary Material 1 [file 12879_2023_8714_MOESM1_ESM.pdf]

Supplementary Tables and Figures for "Comparison of the Clinical Characteristics of SARS-CoV-2 Delta (B.1.617.2) and Omicron (B.1.1.529) Infected Patients from a Single Hospitalist Service"

Table S1. Frequency (percent) for qualitative and median (IQR) for quantitative characteristics of unvaccinated patients infected by Omicron or Delta who were admitted to the University of Florida Health System during Jun. 2021 – Feb. 2022. q-values≤0.05 (bolded) control the false discovery rate to be ≤5%.

Characteristics	Category	Delta	Omicron	p-value	q-value
Total		200	208		
Symptoms	Fever	112 (56.00)	66 (31.73)	<0.001	**<0.001**
	Chills	72 (36.00)	48 (23.08)	0.005	**0.026**
	Stuffy nose	28 (14.00)	27 (12.98)	0.774	0.837
	Runny nose	13 (6.50)	18 (8.65)	0.458	0.588
	Sore throat	13 (6.50)	32 (15.38)	0.004	**0.026**
	Loss of Taste and Smell	14 (7.00)	3 (1.44)	0.006	**0.026**
	Earache	1 (0.50)	0 (0.00)	0.490	0.613
	Headache	28 (14.00)	26 (12.50)	0.664	0.772
	Cough	128 (64.00)	117 (56.25)	0.129	0.224
	Sputum production	32 (16.00)	24 (11.54)	0.199	0.316
	Shortness of Breath	130 (65.00)	108 (51.92)	0.009	**0.034**
	Fatigue	97 (48.50)	94 (45.19)	0.552	0.673
	Myalgias	60 (30.00)	52 (25.00)	0.269	0.408
	Loss of Appetite	54 (27.00)	44 (21.15)	0.202	0.316
	Nausea	55 (27.50)	54 (25.96)	0.738	0.837
	Vomiting	32 (16.00)	36 (17.31)	0.791	0.837
	Diarrhea	57 (28.50)	51 (24.52)	0.372	0.502
	Abdominal pain	16 (8.00)	33 (15.87)	0.015	**0.049**
	No Symptoms	7 (3.50)	10 (4.81)	0.623	0.741
	Other	37 (18.50)	40 (19.23)	0.900	0.900
	Confusion, altered mental status	13 (6.50)	20 (9.62)	0.279	0.410

CXR or Chest CT	Clear	36 (18.00)	81 (38.94)	<0.001	**<0.001**
	Unilateral infiltrate	15 (7.50)	26 (12.50)		
	Multifocal infiltrates	145 (72.50)	96 (46.15)		
	ARDS (ext. pulmonary edema)	0 (0.00)	0 (0.00)		
Severity	Mild	32 (16.00)	70 (33.65)	<0.001	**<0.001**
	Moderate	39 (19.50)	42 (20.19)		
	Severe	100 (50.00)	88 (42.31)		
	Critical	29 (14.50)	8 (3.85)		
Level of Care	Medical/Surgical Floor	134 (67.00)	149 (71.63)	0.123	0.224
	IMC	43 (21.50)	47 (22.60)		
	MICU	23 (11.50)	12 (5.77)		
Days from Onset to Admission		7 (3, 10)	3 (2, 7)	<0.001	**<0.001**
Days from PCR to Admission		0 (0, 1)	0 (0, 0)	0.016	**0.049**
Baseline Clinical Measurements	O2 Saturation	92.00 (88.00, 95.00)	94.00 (90.00, 96.00)	<0.001	**0.003**
	Respiratory Rate	22.50 (18.00, 28.00)	20.00 (18.00, 24.00)	0.001	**0.009**
	NEWS2 Score	5.00 (3.75, 7.25)	5.00 (2.00, 7.00)	0.073	0.147
	WBC	6100 (4500, 8925)	7000 (4950, 9300)	0.130	0.224
	Neutrophils	77.60 (69.05, 83.15)	74.75 (64.00, 82.65)	0.024	0.064
	Lymphocytes	800 (540, 1187.5)	892.5 (600, 1360)	0.068	0.146
	Creatinine	0.94 (0.75, 1.23)	1.00 (0.76, 1.44)	0.297	0.425
	CRP	68.59 (27.27, 129.0)	48.28 (17.98, 121.97)	0.033	0.079
	Procalcitonin	0.09 (0.05, 0.21)	0.11 (0.05, 0.29)	0.175	0.292
	Baseline Rothman Score	74.00 (60.75, 84.00)	69.00 (50.00, 80.00)	0.007	**0.029**
	D dimer	0.97 (0.58, 1.75)	1.04 (0.64, 1.98)	0.452	0.588
Management	IV fluids	53 (26.50)	65 (31.25)	0.326	0.453
	Antibiotics	90 (45.00)	75 (36.06)	0.070	0.146
	Nasal O2	91 (45.50)	71 (34.13)	0.020	0.056
	High flow O2	50 (25.00)	29 (13.94)	0.006	**0.026**
	Remdesivir	121 (60.50)	146 (70.19)	0.048	0.109
	Dexamethasone	143 (71.50)	110 (52.88)	<0.001	**0.002**

	IL6 inhibitor	10 (5.00)	2 (0.96)	0.019	0.055
	Other	12 (6.00)	26 (12.50)	0.027	0.067
	Anticoagulation	143 (71.50)	171 (82.21)	0.013	**0.047**
Death (All cause)	In hospital	9 (4.50)	11 (5.29)	0.820	0.837
	In hospital or ≤30 days of discharge	12 (6.00)	22 (10.58)	0.108	0.208
Death (COVID)	In hospital	7 (3.50)	9 (4.33)	0.800	0.837
	In hospital or ≤30 days of discharge	9 (4.50)	11 (5.29)	0.820	0.837

Table S2. Logistic regression analysis of deaths among hospitalized patients infected by the Omicron and Delta Variants, where Omicron patients are restricted those who were sequence-confirmed. Showing Odds Ratios† (95% confidence intervals).

Variables	COVID-attributed Deaths		All-Cause Deaths	
	In-hospital	In-hospital + ≤30 days of discharge	In-hospital	In-hospital + ≤30 days of discharge
Age group (>= 60 vs. <60)	7.05 (1.74, 48.03)	3.71 (1.24, 13.78)	9.75 (2.47, 65.75)	4.11 (1.53, 13.19)
Immunocompromised (Yes vs. No)	3.39 (0.74, 13.25)	1.98 (0.48, 6.67)	3.40 (0.76, 12.94)	2.21 (0.63, 6.60)
Diabetes Mellitus (Yes vs. No)	1.39 (0.35, 4.72)	1.11 (0.34, 3.18)	2.19 (0.68, 6.60)	1.94 (0.79, 4.57)
Hypertension (Yes vs. No)	0.42 (0.12, 1.30)	0.51 (0.19, 1.33)	0.40 (0.12, 1.19)	0.66 (0.28, 1.51)
Other underlying diseases (Yes vs. No)	1.18 (0.36, 3.62)	1.75 (0.69, 4.45)	0.99 (0.31, 2.87)	1.76 (0.78, 3.97)
Reason of admission (Other vs. COVID-19)	0.00 (-)	0.19 (0.03, 0.71)	0.30 (0.05, 1.14)	0.64 (0.24, 1.54)
Positive RT-PCR test before admission‡ (Yes vs. No)	1.99 (0.65, 5.95)	1.20 (0.43, 3.12)	2.91 (1.03, 8.32)	1.67 (0.69, 3.90)
Variant (Delta vs. Omicron)	0.96 (0.32, 3.13)	0.66 (0.26, 1.71)	1.22 (0.43, 3.84)	0.60 (0.27, 1.37)
Vaccinated (≥1 vs. 0 doses)	1.39 (0.40, 4.38)	1.40 (0.50, 3.69)	1.27 (0.37, 3.83)	1.11 (0.43, 2.66)

† Odds Ratios stratified by whether death was attributed to COVID-19 and whether death occurred during hospitalization. Results with statistical significance are bolded.

‡ Positive RT-PCR test before admission vs. after admission correspond to days from RT-PCR+ to admission >0 vs. ≤0.

Table S3. Odds ratios (95% confidence interval) based on logistic regression of death outcome among hospitalized patients infected with delta or omicron, stratified by whether death was attributed to COVID-19 and whether death occurred during hospitalization. Backward variable selection was conducted for each outcome separately. Variant and vaccination status were forced to stay in each model.

Variables	COVID-attributed Deaths		All-Cause Deaths	
	In-hospital	In-hospital + ≤30 days of discharge	In-hospital	In-hospital + ≤30 days of discharge
Age group (>= 60 vs. <60)	12.67 (3.63, 80.22)	8.01 (3.06, 27.58)	18.36 (5.32, 115.98)	8.69 (3.72, 25.43)
Immunocompromised (Yes vs. No)			2.97 (1.11, 7.40)	2.32 (1.00, 5.06)
Diabetes Mellitus (Yes vs. No)			2.15 (1.01, 4.50)	
Hypertension (Yes vs. No)	0.38 (0.17, 0.81)	0.42 (0.21, 0.82)	0.37 (0.18, 0.77)	
Reason of admission (Other vs. COVID-19)	0.11 (0.02, 0.39)	0.28 (0.10, 0.64)		
Positive RT-PCR test before admission* (Yes vs. No)			2.26 (1.08, 4.64)	
Variant (delta vs. omicron)	0.78 (0.33, 1.75)	0.65 (0.30, 1.34)	0.88 (0.40, 1.86)	0.62 (0.32, 1.15)
Vaccinated (≥1 vs. 0 doses)	1.77 (0.81, 3.83)	1.41 (0.71, 2.77)	1.37 (0.65, 2.84)	1.13 (0.63, 2.02)

*Positive RT-PCR test before admission vs. after admission correspond to days from RT-PCR+ to admission >0 vs. ≤0.

Table S4. Odds ratios (95% confidence interval) based on logistic regression of death outcome among hospitalized patients infected with omicron, stratified by whether death was attributed to COVID-19 and whether death occurred during hospitalization. Backward variable selection was conducted for each outcome separately. Vaccination status was forced to stay in each model.

Variables	COVID-attributed Deaths		All-Cause Deaths	
	In-hospital	In-hospital + ≤30 days of discharge	In-hospital	In-hospital + ≤30 days of discharge
Age group (>= 60 vs. <60)	14.15 (2.75, 260.34)	9.06 (2.58, 57.51)	20.40 (4.09, 371.93)	10.27 (3.52, 44.05)
Immunocompromised (Yes vs. No)	3.63 (1.08, 11.36)		3.92 (1.33, 10.81)	3.05 (1.19, 7.53)
Hypertension (Yes vs. No)	0.37 (0.14, 0.96)	0.41 (0.18, 0.91)	0.40 (0.17, 0.94)	
Other underlying diseases (Yes vs. No)				2.48 (1.28, 4.90)
Reason of admission (Other vs. COVID-19)	0.16 (0.02, 0.58)	0.37 (0.13, 0.90)		
Vaccinated (≥1 vs. 0 doses)	1.59 (0.59, 4.34)	1.56 (0.71, 3.47)	1.60 (0.68, 3.87)	1.15 (0.58, 2.26)

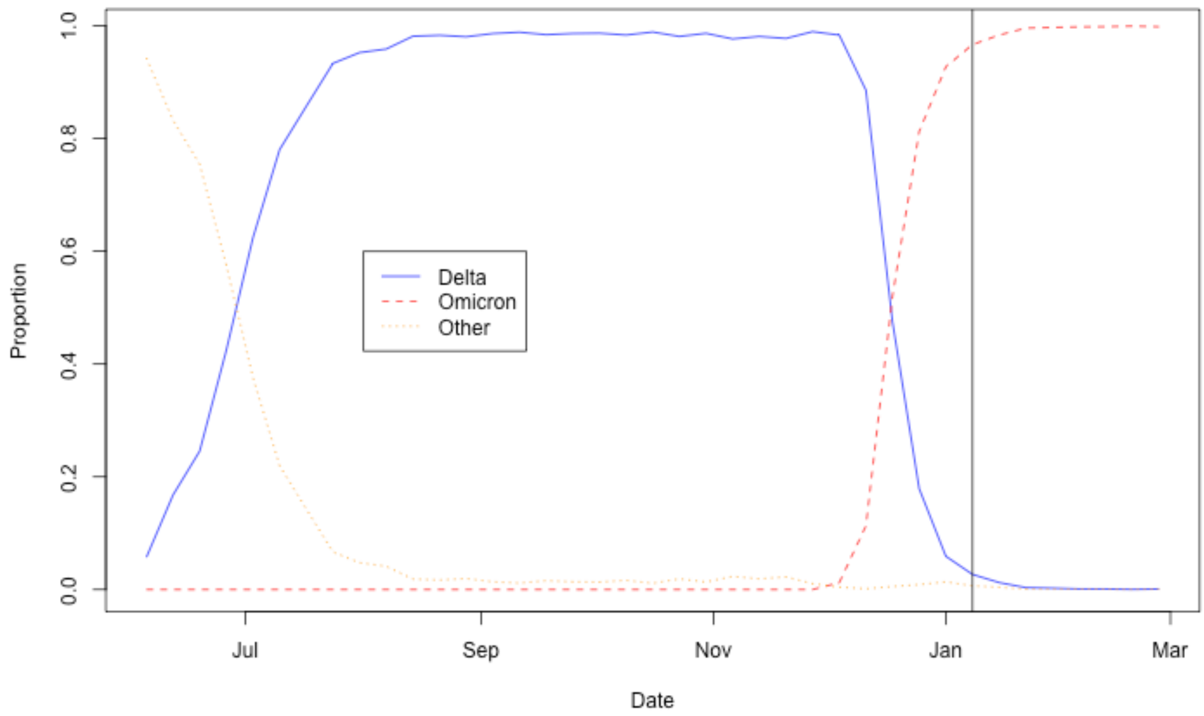

Figure S1: Proportion of variants in the southeastern region (Alabama, Florida, Georgia, Kentucky, Mississippi, North Carolina, South Carolina, Tennessee) during June 1, 2021 – March 1, 2022. The vertical solid line marks January 8, 2022. Data source: (https://covid.cdc.gov/covid-data-tracker/#variant-proportions).

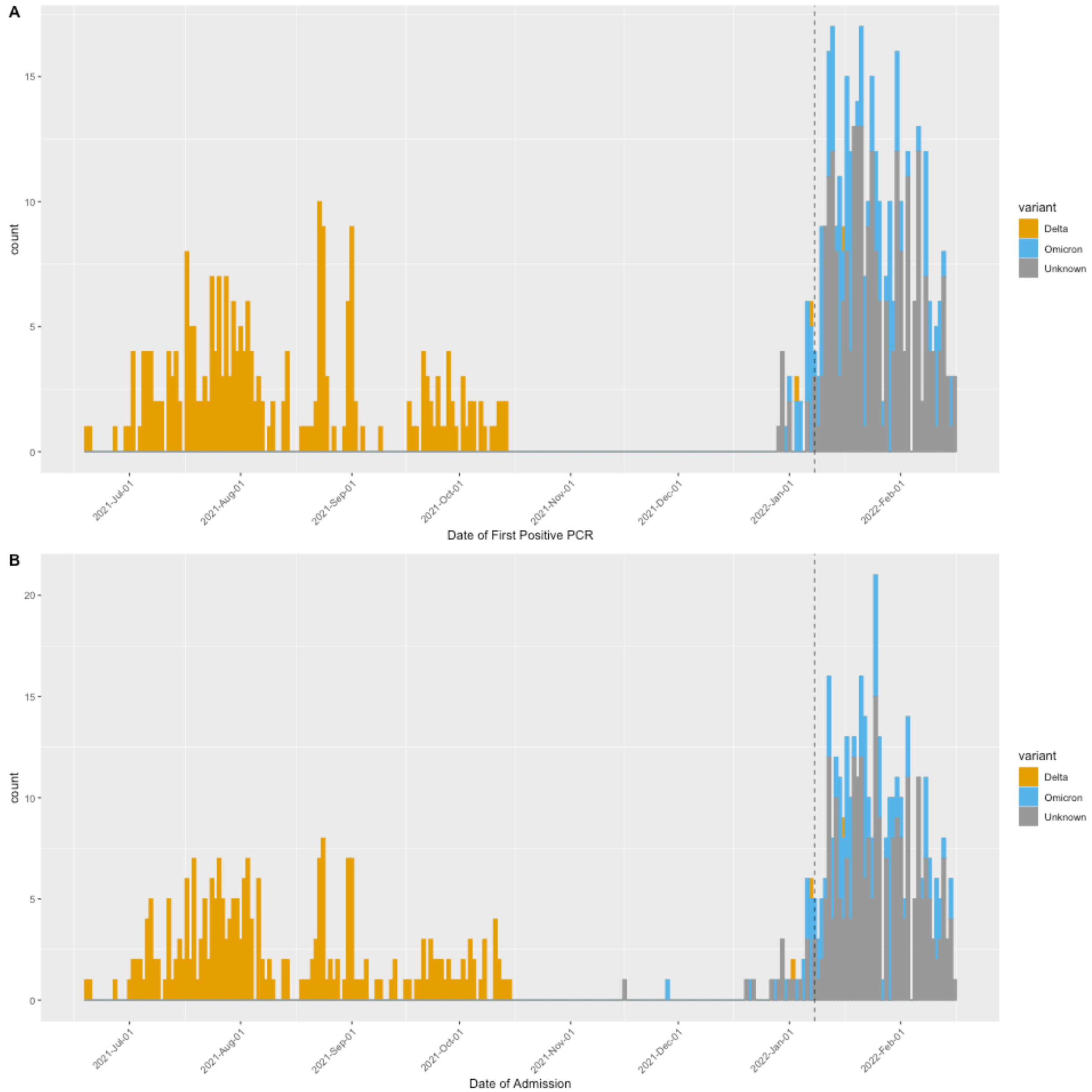

Figure S2: Timeline of patients admitted during June 2021 – February 2022 by date of first positive PCR (A) and date of admission (B). Patients detected after November 2021 and confirmed by whole genome sequencing were colored in blue for Omicron and orange for Delta. Patients detected before November 2021 were suspected to be Delta-infected and also colored in orange. Patients who were detected after November 2021 but had no whole genome sequence were colored in grey. The vertical solid line marks January 8, 2022, after which we assume all patients without whole genome sequence were Omicron-infected.

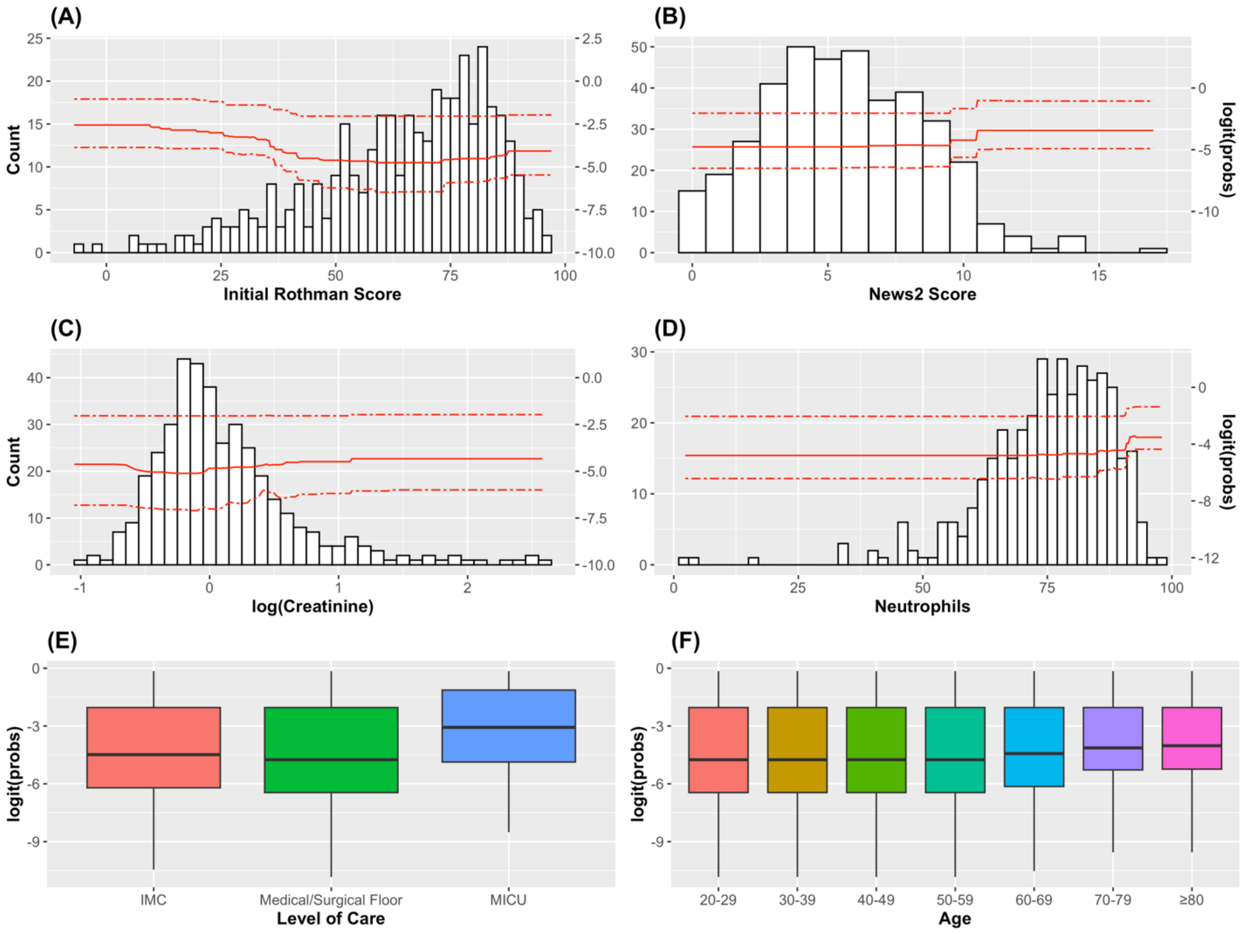

Figure S3: Average (solid red) and (2.5%, 95%) quantiles of response curves over the 100 random data splits for factors with relative importance ≥7 based on the XGBOOST model for predicting in-hospital death of all causes: (A) Initial Rothman Score, (B) NEW2 Score, (C) log(Creatinine), (D) Neutrophils, (E) Level of Care, and (F) Age group. In (A)-(D), the background histograms show the distribution of the factor’s values in the data.

Supplementary Methods

Criteria for Hospital Admission

Positive SARS-CoV-2 positive RTPCR

AND

an increased respiratory rate (≥30 breaths per min)

OR

hypoxia with oxygen saturation ≤ 94% on RA or a decrease in saturation to < 90% with ambulation.

OR

In the absence of the above findings, patients with a high risk of a poor outcome:

- Age 65 years or older
- Serious cardiovascular disease including heart failure, coronary artery disease, cardiomyopathy
- Chronic obstructive pulmonary disease
- Diabetes
- Active malignancy
- Obesity (BMI ≥30 kg/m2)
- Chronic kidney disease
- Immunocompromised state with solid organ transplantation and other patients receiving immunosuppressive therapy
- Pregnancy
- Sickle cell disease.

WHO classification of illness severity (12/2022, included on the Red Cap entry form below)
Mild Illness*:* Individuals who have any of the various signs and symptoms of COVID-19 but who do not have shortness of breath, dyspnea, or abnormal chest imaging.
Moderate Illness*:* Individuals who show evidence of lower respiratory disease during clinical assessment or imaging and who have an oxygen saturation (SpO2) ≥94% on room air at sea level.
Severe Illness*:* Individuals who have SpO2 < 94% on room air at sea level, a ratio of arterial partial pressure of oxygen to fraction of inspired oxygen (PaO2/FiO2) < 300 mm Hg, respiratory frequency >30 breaths/min, or lung infiltrates >50%.
Critical Illness*:* Individuals who have respiratory failure, septic shock, and/or multiple organ dysfunction.

Criteria for assigning the cause of death to be COVID-19

- The reason for admission was determined to be COVID-19
- Laboratory data indicated active SARS-COV-2 infection – oxygen saturation < 94%, , low lymphocyte count, elevated CRP
- Infiltrate on CXR or pulmonary CT scan.
- COVID-19 listed as a primary diagnosis on the discharge summary.
- No other underlying illness was likely to be the cause of death.

Additional Details on X-Boost Machine Learning Risk of Death Model

A tree complexity of five, a learning rate of 0.005 and a bagging fraction of 75% were used based on our previous research. A 10-fold cross validation was used to identify the optimal number of trees using the *gbm.step* function. To improve predictive power, we randomly divided the data set into 75% training set and 25% test set for 100 times to form 100 permutation data sets and fitted BRT to each data set. The outputs of the BRT models, both marginal effect curves and relative contributions of predictors, were summarized by median and inter-quartile range over the 100 permutation data sets. For each model, the relative contribution was calculated based on how many times a predictor was chosen for splitting and how much each split improved the objective function, averaging over all model-included trees. Modeling analyses was performed for all deaths and COVID-19-attributable deaths separately. In addition, a significant number of deaths occurred after discharge, and modeling analyses was also stratified by whether deaths within 30 days of discharge were included as death events. Deaths beyond 30 days of discharge were considered non-death events.

Figure S4 Sample RedCap Entry Form

Omicron Case Description Form

Record ID	__________________________
Medical Record Number	__________________________
Patient's age	◯ 20-29 years<br>◯ 30-39 years<br>◯ 40-49 years<br>◯ 50-59 years<br>◯ 60-69 years<br>◯ 70-79 years<br>◯ 80 years or older
Sex	◯ Male<br>◯ Female
Vaccinated for SARS-CoV2	◯ No<br>◯ Yes 1 shot<br>◯ Yes 2 shots<br>◯ Yes 3 shots
Days since first symptom	◯ 1 day<br>◯ 2 days<br>◯ 3 days<br>◯ 4 days<br>◯ 5 days<br>◯ 6 days<br>◯ 7 days<br>◯ 10 days<br>◯ 14 days<br>◯ 21 days<br>◯ 28 days<br>◯ > 28 days
Date RTPCR positive test	__________________________
Interval Positive RTPCR to Admission	__________________________
Variant Type	◯ Omicron B1<br>◯ Omicron B2<br>◯ Delta<br>◯ Other

Major Symptoms	☐ Fever<br>☐ Chills<br>☐ Stuffy nose<br>☐ Runny nose<br>☐ Sore throat<br>☐ Loss of Taste and Smell<br>☐ Earache<br>☐ Headache<br>☐ Cough<br>☐ Sputum production<br>☐ Shortness of Breath<br>☐ Fatigue<br>☐ Myalgias<br>☐ Loss of Appetite<br>☐ Nausea<br>☐ Vomiting<br>☐ Diarrhea<br>☐ Abdominal pain<br>☐ Confusion, altered mental status<br>☐ No Symptoms<br>☐ Other
Other Symptoms	__________________________________
Comments about present illness: Timing of symptoms if not simultaneous, Other medical problem that explains the reason for admission.	__________________________________
Underlying disorders	☐ Cardiovascular Disease<br>☐ Hypertension<br>☐ Diabetes Mellitus<br>☐ COPD<br>☐ Obesity<br>☐ Sickle Cell Disease<br>☐ Asthma<br>☐ Active Cancer<br>☐ Immunocompromised<br>☐ Other
Other underlying diseases	__________________________________
O2 sat RA	__________________________________
Respiratory Rate (highest day of admission)	__________________________________
NEWS2 Score (use MD Calc)	__________________________________
White Blood Cell Count (cells/µL) (Example 4,500)	__________________________________
Laboratory procedure percent neutrophils value	__________________________________

Laboratory Procedure: total lymphocyte count (cells/µL) (Example 1500)	____________________
Laboratory procedure creatinine value	____________________
C-reactive Protein (CRP) Result (mg/L)	____________________
D-dimer Result Value	____________________
Procalcitonin	____________________
Lab Miscellaneous	____________________
CXR or Chest CT	◯ Clear<br>◯ Unilateral infiltrate<br>◯ Multifocal infiltrates<br>◯ ARDS (extensive pulmonary edema)
Comments about chest imaging	____________________
COVID-19 Severity	◯ Mild<br>◯ Moderate<br>◯ Severe<br>◯ Critical<br>(Mild Illness: Individuals who have any of the various signs and symptoms of COVID-19 (e.g., fever, cough, sore throat, malaise, headache, muscle pain, nausea, vomiting, diarrhea, loss of taste and smell) but who do not have shortness of breath, dyspnea, or abnormal chest imaging. Moderate Illness: Individuals who show evidence of lower respiratory disease during clinical assessment or imaging and who have an oxygen saturation (SpO2) ≥94% on room air at sea level. Severe Illness: Individuals who have SpO2 < 94% on room air at sea level, a ratio of arterial partial pressure of oxygen to fraction of inspired oxygen (PaO2/FiO2) < 300 mm Hg, respiratory frequency >30 breaths/min, or lung infiltrates >50%. Critical Illness: Individuals who have respiratory failure, septic shock, and/or multiple organ dysfunction.)
Level of Care	◯ Medical/Surgical Floor<br>◯ IMC<br>◯ MICU
Reason for Admission	◯ COVID-19<br>◯ Other
Comments on reason for admission	____________________

Management Plan	☐ IV fluids<br>☐ Antibiotics<br>☐ Oxygen supplement - nasal O2<br>☐ Oxygen supplement - high flow O2<br>☐ Remdesivir<br>☐ Dexamethasone<br>☐ IL6 inhibitor<br>☐ Anticoagulation<br>☐ Other
If antibiotics administered, who ordered?	☐ ED<br>☐ MHS<br>☐ ED and MHS
Anticoagulation	☐ Heparin prophylactic dose<br>☐ Heparin therapeutic dose<br>☐ Lovenox prophylactic dose<br>☐ Lovenox therapeutic dose
Other treatments:	______________________________
Date of Admission	______________________________
Date of Discharge	______________________________
Length of hospital stay	______________________________
Hospital Course - Rothman Index. Look at the scores the day of positive RTPCR test or if the test was before admission on the day of admission, and look at the scores the day of discharge. If the discharge score is 10 points or higher than the admission score = Improved. 10 points lower = Worsened. If no < 10 point change = No significant change. (Access RI trend graph by opening the chart through Patient Station)	☐ Improved<br>☐ No significant change<br>☐ Worsened
Rothman Index at the time RTPCR positive (closest date)	______________________________
Rothman index at the time of discharge	______________________________
Rothman Change	______________________________
Died in Hospital	◯ Yes<br>◯ No
Cause of Death COVID	◯ Yes<br>◯ No
Died after discharge	◯ Yes<br>◯ No

Date of Death after discharge

____

Interval from discharge to death

____
